# A comparison of rule-based and centroid single-sample multiclass predictors for transcriptomic classification

**DOI:** 10.1093/bioinformatics/btab763

**Published:** 2021-11-12

**Authors:** Pontus Eriksson, Nour-al-dain Marzouka, Gottfrid Sjödahl, Carina Bernardo, Fredrik Liedberg, Mattias Höglund

**Affiliations:** Division of Oncology, Department of Clinical Sciences, Lund University, Lund, Sweden; Division of Oncology, Department of Clinical Sciences, Lund University, Lund, Sweden; Urology - urothelial cancer, Department of Translational Medicine, Lund University, Skåne University Hospital, Malmö, Sweden; Division of Oncology, Department of Clinical Sciences, Lund University, Lund, Sweden; Urology - urothelial cancer, Department of Translational Medicine, Lund University, Skåne University Hospital, Malmö, Sweden; Division of Oncology, Department of Clinical Sciences, Lund University, Lund, Sweden

## Abstract

**Motivation:**

Gene expression-based multiclass prediction, such as tumor subtyping, is a non-trivial bioinformatic problem. Most classifier methods operate by comparing expression levels relative to other samples. Methods that base predictions on the expression pattern within a sample have been proposed as an alternative. As these methods are invariant to the cohort composition and can be applied to a sample in isolation, they can collectively be termed single sample predictors (SSP). Such predictors could potentially be used for preprocessing-free classification of new samples and be built to function across different expression platforms where proper batch and dataset normalization is challenging. Here, we evaluate the behavior of several multiclass SSPs based on binary gene-pair rules (k-Top Scoring Pairs, Absolute Intrinsic Molecular Subtyping and a new Random Forest approach) and compare them to centroids built with centered or raw expression values, with the criteria that an optimal predictor should have high accuracy, overcome differences in tumor purity, be robust across expression platforms and provide an informative prediction output score.

**Results:**

We found that gene-pair-based SSPs showed excellent performance on many expression-based classification tasks. The three methods differed in prediction score output, handling of tied scores and behavior in low purity samples. The k-Top Scoring Pairs and Random Forest approach both achieved high classification accuracy while providing an informative prediction score. Although gene-pair-based SSPs have been touted as being cross-platform compatible (through training on mixed platform data), out-of-the-box compatibility with a new dataset remains a potential issue that warrants cohort-to-cohort verification.

**Availability and implementation:**

Our R package ‘multiclassPairs’ (https://cran.r-project.org/package=multiclassPairs) (https://doi.org/10.1093/bioinformatics/btab088) is freely available and enables easy training, prediction, and visualization using the gene-pair rule-based Random Forest SSP method and provides additional multiclass functionalities to the switchBox k-Top-Scoring Pairs package.

**Supplementary information:**

Supplementary data are available at *Bioinformatics* online.

## 1 Introduction

Tumors originating from the same tissue can have highly disparate molecular characteristics. This molecular heterogeneity has motivated efforts to stratify cancer into molecular categories based on differences in mRNA expression, protein expression, methylation patterns, gene mutations or copy number alterations. Gene expression-based stratifications provide a way to systematize biological and clinical research, and has the potential to improve prognostication and therapy prediction. To utilize such stratifications, a prediction method must be constructed that accurately calls the class of new samples. Centroid-based classification methods that use an idealized profile (centroid) of selected genes whose expression differ between subtypes are commonly used. A new sample is classified by determining which centroid it most closely resembles, as measured by correlation or other similarity measure. Centroid variations such as Prediction Analysis of Microarrays (PAM) (Tibshirani *et al.*, 2002) and Classification to Nearest Centroids (ClaNC) (Dabney, 2005) advance this concept by utilizing shrunken centroids or class-specific genes, respectively. A drawback of centroid methods is that they conventionally rely on log-transformed expression data row-centered across samples (relative fold change values) which can be impacted by the cohort composition (*e.g.,* proportion of tumor stages or subtypes). Before such a classifier can be used, a new dataset needs to be processed and normalized to ensure that it conforms to the expected data distribution. Rule-based classifiers have been proposed as an alternative approach where non-cohort-normalized expression data (such as array signal intensities or gene-length normalized RNA-sequencing counts) is converted into binary gene-pair rules. The concept is that characteristic gene ratios can be used to identify different subtypes. A gene-pair decision rule can be described as ‘if Gene A > Gene B is TRUE this indicates Class X, else non-Class X’. Although information carried by continuous expression values is lost when the data is featurized, these binary rules are not dependent on between-sample normalization making the classifier applicable to samples in isolation *i.e.,* a single sample predictor (SSP). The featurizing also enables data from different platforms and preprocessing pipelines to be used during training to find class specific gene ratios captured across methods in order to create a more broadly applicable classifier, as demonstrated in earlier works (Cirenajwis *et al.*, 2020; Paquet and Hallett, 2015). Geman *et al.* first described a two-class predictor using a single gene-pair rule, referred to as a Top Scoring Pair (TSP) (Geman *et al.*, 2004), which was expanded into the k-Top Scoring Pairs (k-TSP) method where multiple rules vote on the class labels (Tan *et al.*, 2005). Gene-pair-based methods can be extended to multiclass prediction problems using multiple binary classifiers or by combining informative gene-pair rules with methods compatible with multiclass prediction. Some proposed methods include votes from 1-versus-rest or 1-versus-1 k-TSP classifiers, hierarchical application of k-TSP classifiers (Tan *et al.*, 2005), TSP decision trees (Popovici *et al.*, 2011) or using preselected rules in a Naïve Bayes (NB) classifier (AIMS) (Paquet and Hallett, 2015). Here, we evaluate two k-TSP classifier R implementations (AIMS and switchBox) and develop a new approach utilizing Random Forest (RF).

We apply these methods to bladder cancer subtype prediction and compare them to a currently utilized single sample centroid approach (Kamoun *et al.*, 2020; Lindskrog *et al.*, 2021). The creation of a robust subtype prediction model for bladder cancer is challenging both due to the molecular heterogeneity of the disease, but also by technical circumstances resulting in major batch effects between datasets. Large gene expression cohorts (>200 samples) remain scarce and are generally not population based. Most datasets are constructed based on tumor stage, representing either non-muscle invasive or muscle invasive disease, with varying subtype distributions due to sample inclusion criteria. There is also variation in tissue sources *e.g.,* fresh frozen or FFPE, as well as mRNA quantification methods, and sample purity can vary broadly both due to intratumor immune and stroma levels or adjacent normal tissue in the biopsy. These factors, taken together, are challenging to overcome with standard batch correction methods and can strongly impact prediction results of a conventional centroid-based classifier. This has hindered robust validation efforts and confident adoption of any one tumor classification scheme proposed for the disease, which motivated us to evaluate alternative classifier methods that could overcome these problems.

## 2 Materials and methods

### 2.1 Bladder cancer datasets

We utilized two bladder cancer dataset, an in-house Affymetrix microarray dataset of 301 tumors (Lund2017) (GSE83586) (Sjödahl *et al.*, 2017) and the TCGA-BLCA bladder cancer RNA-sequencing dataset of 397 tumors (TCGA) (https://gdc.cancer.gov) (Robertson *et al.*, 2017). Both datasets are similar in stage composition, containing advanced and muscle invasive tumors. When classifiers were trained on the Lund2017 dataset the TCGA was used as validation, and vice versa, to evaluate a ‘single training dataset’ scenario. To evaluate training on mixed data, the cohorts were combined, and five cross-validation data splits were sampled, using 80% of samples for training and 20% for testing (Lund2017/TCGA_cv1-5). A 5-group subtype split based on the Lund2017 taxonomy was used as reference labels (Supplementary Fig. S1), obtained from previous studies of these cohorts (Marzouka *et al.*, 2018; Sjödahl *et al.*, 2017). Tumors are classified as either Urothelial-like (Uro), Genomically Unstable (GU), Basal/Squamous-like (Ba/Sq), Mesenchymal-like (Mes-like) or Small cell/Neuroendocrine-like (Sc/NE). The Urothelial-like subtype can be further stratified into UroA, UroB and UroC which share many transcriptional programs but differ in growth patterns and mutational makeup (Marzouka *et al.*, 2018; Sjödahl *et al.*, 2017). The Lund2017 cohort stratification was generated through hierarchical clustering to determine the major transcriptional classes, followed by curation through immunostaining where highly infiltrated samples are resolved into their respective tumor cell phenotype class. Without curation, highly infiltrated tumors tend to form a separate cluster when unsupervised gene expression clustering is used (Aran *et al.*, 2015; Rhee *et al.*, 2018; Sjödahl *et al.*, 2017). The TCGA-BLCA labels were assigned using a centroid classifier based on the granular stratification of the Lund2017 cohort, with separate centroids for high and low infiltration categories for each subtype (Marzouka *et al.*, 2018).

### 2.2 Additional datasets

A total of 1084 samples from 18 bladder cancer datasets was used to further examine cross-platform performance (Kamoun *et al.*, 2020). A cohort of 3814 breast cancer samples from the Swedish Cancerome Analysis Network—Breast (SCANB) was used to evaluate PAM50 subtype prediction in a population-based RNA-sequencing dataset in log2(FPKM + 0.1) format (GSE81538 + GSE96058) (Brueffer *et al.*, 2018). A meta-cohort of 19 lung cancer datasets was used to test histology and subtype prediction in a heterogeneous cohort generated on diverse platforms (Supplementary Table S1). This dataset has previously been used to evaluate single sample prediction methods (Cirenajwis *et al.*, 2020) and was provided by the authors. The PAN-CANCER TCGA dataset was used to test tumor-type prediction, representing a classification problem with a high number of distinct classes (Liu *et al.*, 2018). The dataset of 10 088 samples representing 33 tumor types was obtained through the Recount2 R package in TPM format (Collado-Torres *et al.*, 2017).

### 2.3 Centroid classifiers

#### 2.3.1 Single sample centroid classifier

Centroids built on log-transformed raw expression data have recently been utilized as SSPs for bladder cancer subtyping (Kamoun *et al.*, 2020; Lindskrog *et al.*, 2021), and similar approaches have been explored for other cancers (Hu *et al.*, 2006; Sorlie *et al.*, 2003). We built and applied the single sample centroids (SS-centroids) following the method reported for bladder cancer, selecting differentially expressed genes on a per-subtype basis using moderated t-test (*P*-value < 0.05) and a ‘Subtype vs Rest’ AUC above 0.6, followed by ranking on mean fold change. Log2(TPM + 1) data was used for the TCGA dataset and log2(RMA probe values, median merged to gene symbols) was used for the Lund2017 dataset. The mean raw expression of the selected genes for each subtype was used as centroids. Pearson correlation was used as distance metric (1-Pearson's r) to determine the nearest centroid for each sample. We evaluated centroids of increasing sizes by gradually including more genes per class based on the Subtype versus Rest gene ranking. The smallest centroid used the top 10 upregulated and 10 downregulated genes per class, while the largest used 125 up- and downregulated genes per class, resulting in centroids ranging from 67 to 774 genes. Training accuracy is reported by a direct reapplication of the centroids to the training data.

#### 2.3.2 Conventional nearest centroid classifier

We compared the raw data single sample centroids to centroids built on centered log-transformed data. Batch-corrected median-centered log2-transformed data was used for Lund2017 data, and centered VST-transformed counts was used for TCGA data. The gene selection applied to centered data resulted in gene lists highly similar to those of the single sample centroids. As we were primarily interested in predictor behavior differences between raw data and centered data, we opted to use the same centroid genes as selected for the respective single sample centroids but calculating the values from the centered data. Pearson correlation was again used as distance metric.

### 2.4 Rule-based classifiers

#### 2.4.1 Aims — k-TSP using Naïve Bayes

The code for Absolute Intrinsic Molecular Subtyping (AIMS) was obtained from Github (https://github.com/meoyo/trainAIMS) (Paquet and Hallett, 2015). AIMS uses all input genes to produce a list of each possible gene-pair rule combination. Scores for all gene-pair rules are calculated in a one-versus-rest fashion for each class (Geman *et al.*, 2004). The optimal number of rules (k) per class is determined by the peak average overall accuracy from a 20-fold cross-validation training step, using the same number of rules for each class. The final classifier selects k rules that are then used in a multi-class NB model. To handle dataset imbalances, AIMS allow the user to weight datasets during training, aiming to reduce the effect of unequal dataset sizes, and NB utilizes the percentage of each class in its prior probabilities. Models were trained using the default setting where the examined number of rules (k) ranged between 1 and 50, using 20-fold cross-validation and weights based on the input training datasets.

#### 2.4.2 switchBox — k-TSP using classifier votes

The k-TSP classifier R package switchBox (SB) was installed from Bioconductor (https://www.bioconductor.org/) (Afsari *et al.*, 2015). SwitchBox builds binary k-TSP classifiers in three steps: gene selection, gene-pair rule scoring, and selection of the optimal number of rules. With default settings, SB uses a one-versus-rest Wilcoxon test to select the top 100 differentially expressed genes (top 50 up- and downregulated) and determines the optimal number of gene-pair rules (ranging between 2 and 10 by default) through variance optimization (Afsari *et al.*, 2014). Gene selection and variance optimization, rather than cross-validation, results in training times of minutes instead of hours (AIMS approach). When used for multi-class prediction a one-versus-rest k-TSP classifier is built for each class to independently generate a score between 0 and 1, where the highest decides the class assignment. Several modifications to the default switchBox k-TSP implementation were evaluated, including increasing the number of used genes and rules, allowing non-differentially expressed ‘pivot genes’ to be paired with the selected genes, using one-versus-one gene selection and rule scoring and performing these steps in a platform-wise manner when multiple datasets were used for training (Supplementary Methods). Reported training accuracies reflects the application of the ensembled classifier to the training dataset.

#### 2.4.3 Rule-based Random Forest

Random Forest (RF) uses an ensemble of decision trees built through randomized feature selection and subsampling (bagging) (Breiman, 2001), which allows multiple different gene-pairs to be utilized through independent decision trees. RF generates a feature importance score during model training, providing a direct way to select genes and gene-pair rules. In our R package multiclassPairs (Marzouka and Eriksson, 2021), we implemented a complete pipeline for creating rule-based predictor utilizing the ‘ranger’ RF R package (Wright and Ziegler, 2017). This builds the predictors in three steps: (i) gene importance ranking and selection through RF, (ii) rule importance ranking and selection through RF and (iii) filtering and selection of rules to be utilized in a final probability-RF classifier predicting all classes. The gene importance ranking was determined by training RF models on ranked raw data to predict all classes at once, from which the highest scoring genes were selected. This gene list was supplemented with top scoring genes from separate one-versus-rest RF models for each class. Genes that score high in the ‘All classes’ model perform well on major splits of the data, while genes identifying smaller classes may be less likely to be among the very top ranking. We evaluated utilizing the top 10, 20, 50, 100 or 200 genes by importance score from each RF model. Unique top genes were combined into binary gene-pair rules. The training of ‘All classes’ and ‘one-vs-rest’ RF models was repeated using rules as training data, giving a variable importance score for each. To diversify the rules and avoid relying on rules with the same informative partner gene, we ranked all rules by their importance for each model separately and applied a filter to retain only the top scoring instances where a given gene is used. The final predictors were built by selecting the top 10, 20, 50, 100 or 200 filtered rules from each classifier and training a final probability-RF model. For gene and rule selection, we used 5000 trees, with ‘node-size’ set to 1, ‘mtry’ set to 10% of features, and ‘impurity’ as variable importance measurement. Variable importance of genes and rules are saved at each modeling step, allowing for examination of the selected features. For the final classifier, ‘mtry’ was set to the square root of the number of rules, using 5000 trees. During model training, ∼37% of samples are randomly left out during the construction of each component decision tree. The ‘Out-of-Bag’ (OOB) training accuracy is measured on the ∼1850 trees where a given sample is excluded from the training. In terms of training time, this approach was slower than most k-TSP variations, but significantly faster than AIMS. To evaluate whether all gene-pair rules selected for the final models were important we utilized the R package Boruta, which compares features against permutated versions of themselves and labels each as confirmed, tentative or rejected (Kursa and Rudnicki, 2010). The Random Forest algorithm does not tolerate absent variables. We addressed this by retaining the binary training matrix as a part of the predictor. When classifying a sample with genes missing, the possible rules are generated and missing ones are imputed with a k-nearest neighbor approach using the modal value from the five most similar training samples (Supplementary Fig. S2).

## 3 Results

### 3.1 Subtype prediction in bladder cancer

We compared how conventional centroids, single-sample centroids, Absolute Intrinsic Molecular Subtyping (AIMS), switchBox k-TSPs and rule-based Random Forest performed on the 5-molecular subtype prediction task in bladder cancer.

#### 3.1.1 Nearest centroid classifiers

**Fig. 1.**
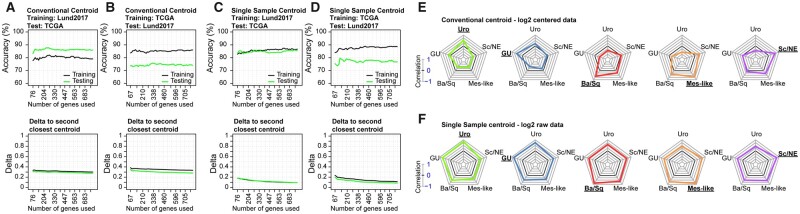
Conventional and single sample centroid behavior. Overall centroid classifier accuracy (top panel) and average correlation difference between the predicted subtype and second closest centroid (bottom panel), trained on centered data from (**A**) Lund2017 and (**B**) TCGA, and raw data from (**C**) Lund2017 and (**D**) TCGA. (**E**) Average sample to centroid correlations for Uro, GU, Ba/Sq, Mes-like and Sc/NE tumors in the Lund2017 dataset using a conventional centroid of 424 genes and (**F**) a single sample centroid of 424 genes

Conventional centroids were trained on the Lund2017 and TCGA datasets separately. The Lund2017 centroids had prediction accuracies ranging between 0.83 and 0.88 when applied to the TCGA data (Fig.  1A) , while those built on TCGA data had prediction accuracies between 0.73 and 0.76 in the Lund2017 data (Fig.  1B). Misclassification events in both the training and test scenarios were dominated by Urothelial-like tumors of the UroB subcategory predicted to be of the Ba/Sq subtype. While UroB tumors retain expression of urothelial differentiation and FGFR3 signaling, shows a stratified growth-pattern and has genomic alterations resembling UroA tumors, they have high expression of numerous keratinization genes in common with the Ba/Sq subtype, resulting in a higher Pearson correlation coefficient to the Ba/Sq centroid (Fig.  4). The single sample (SS) centroid classifiers built using raw expression data from the Lund2017 dataset had training accuracies between 0.83 and 0.87 and prediction accuracies ranging between 0.83 and 0.86 on the TCGA dataset (Fig.  1C). The TCGA SS-centroids had training accuracies between 0.84 and 0.89 and prediction accuracies between 0.74 and 0.80 on the Lund2017 dataset (Fig.  1D). The overall accuracy of both the conventional and single sample centroids remained relatively stable regardless of the number of utilized genes (Fig.  1A–D). The average separation between the predicted class and the second closest prediction remained constant for the conventional centroid but continuously decreased as the single sample centroid size increased. As genes are added based on their ranked fold change, they have a gradually diminished impact on the correlation between samples and the centroids when data is centered. (Fig.  1A, B and E). When uncentered raw data is used, the centroids represent the average gene expression values for each subtype. The correlation is therefore partly driven by the inherent expression ranges of the classifier genes rather than the subtype-specific expression differences, resulting in positive correlations between most samples and the SS-centroids (Fig.  1F). This effect is exacerbated as more genes are added and results in increasingly similar correlations between a sample and any given centroid (Fig.  1C, D and F, Supplementary Fig. S3).

#### 3.1.2 Absolute Intrinsic Molecular Subtyping — AIMS

**Fig. 2.**
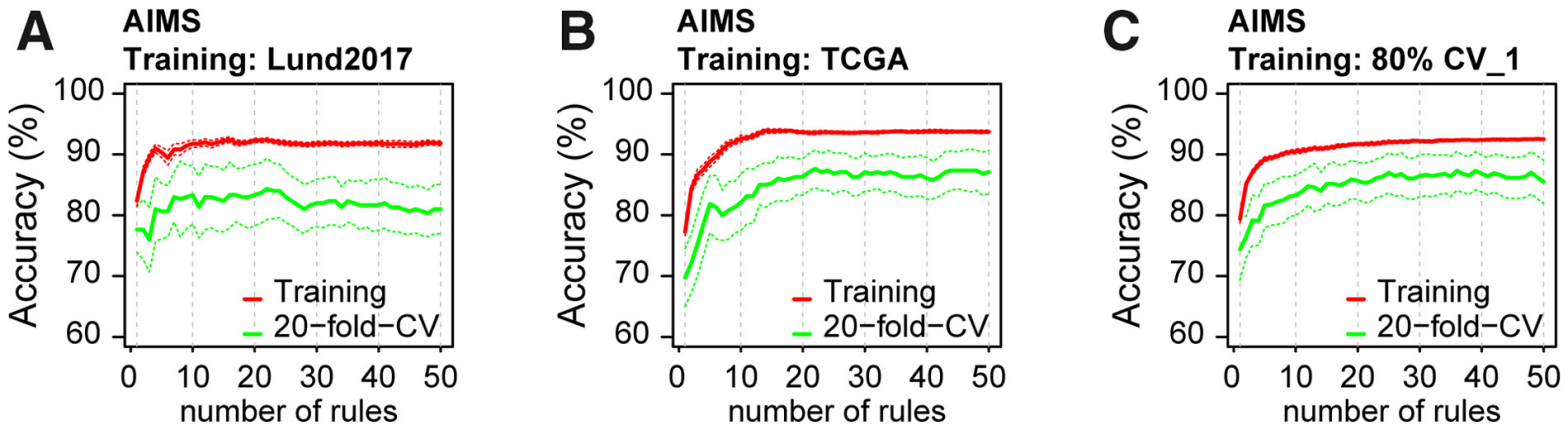
Cross-validation accuracy and overall training accuracy during AIMS model training. The training accuracy of 20-fold CV and direct application on the training data evaluated using between 1 and 50 rules on (**A**) Lund2017, (**B**) TCGA and (**C**) mixed data. The optimal number of rules is determined by the peak 20-fold CV accuracy

The AIMS method was applied with default settings (cv-folds = 20, k range = 2:50 rules per class). The peak average cross-validation accuracy was reached at 22 rules per subtype for both the Lund2017 (0.84) and TCGA (0.87) models (Fig.  2A and B). The Lund2017 model had an accuracy of 0.93 when reapplied to the training data, and a prediction accuracy of 0.73 on the TCGA dataset. The TCGA model had a training accuracy of 0.93, but a low accuracy of 0.53 when applied to the Lund2017 data. The rule-based classifiers trained on isolated datasets contained several rules which were subtype specific in the training data but performed poorly in the test dataset. Rather than failing to indicate the expected subtype, these were commonly TRUE or FALSE across almost the entire test dataset, meaning that the informative expression ratio crossover did not occur on the test platform (Supplementary Fig. S4). Gene pair rules that function across platforms may be identified by training the classifier on mixed data. We built classifiers on five different 80/20 training/test splits of mixed Lund2017/TCGA data. The training accuracy on mixed data was similar to the isolated datasets (Fig.  2C), and the prediction accuracies on the test sets ranged between 0.80 and 0.91 (Supplementary Table S1). Since subtype proportions were similar between the datasets, we calculated AUC values for each rule as a crude measure of platform specificity, which showed that mixed data models included fewer rules with clear platform specific behavior than models trained on isolated datasets (Supplementary Fig. S5). Rules identified on RNA-seq data more commonly perform poorly on the microarray data than vice versa, likely due to the more limited dynamic range of arrays. The benefit of mixing data was seen also when the five training sets were reduced from 555 to 350 samples each, and when the training data proportions were skewed to include 20/80 of samples from the respective platforms (test accuracies of 0.80–0.93).

#### 3.1.3 k-TSP

We used switchBox (SB) to train one-versus-rest k-TSP classifiers for each subtype. The five classifiers were used for multi-class prediction by applying each classifier to a sample and calling the subtype assignment based on the highest individual classifier score. The optimal number of rules was determined independently for each classifier, meaning that a different number of rules may be selected for each class. With default settings, the Lund2017 k-TSP model had a training accuracy of 0.94 and a prediction accuracy of 0.80 on the TCGA dataset, while the TCGA model had a training accuracy of 0.92 and a prediction accuracy of 0.76 on the Lund2017 data (Supplementary Table S1). The models built on mixed training data had prediction accuracies between 0.83 and 0.86 on the five 20% test sets. When the prediction is based on votes from multiple independent classifiers there may be tied scores, particularly if few rules are used. In a two-class problem, this can be avoided using an odd number of rules, but this does not work for multi-class prediction. We evaluated the occurrence of tied scores by forcing the individual subtype classifiers to use a fixed number of rules (ranging from 2 to 50). Tied scores were prevalent when less than 10 rules per class were used, indicating that increasing the number of rules may be warranted for multi-class prediction tasks (Supplementary Fig. S6). Increasing the number of input genes to include the top 500 up- and downregulated and the max number of allowed rules to 50 resulted in modest improvements in prediction accuracies (0.81 for the Lund2017 and 0.84 for the TCGA classifiers). With these settings, switchBox consistently selected a higher number of rules for the final model for most classes, regardless of training dataset (Supplementary Table S1). The classifiers trained on mixed data also reached slightly higher peak prediction accuracy when more genes and rules were allowed, ranging between 0.82 and 0.88 on the test sets. The inclusion and behavior of platform specific rules was similar to that observed for the AIMS models (Supplementary Fig. S5).

We evaluated modifications to the gene and rule selection methods implemented in switchBox, including one-versus-one gene filtering, platform-wise gene filtering, platform-wise rule scoring and one-versus-one rule scoring. For bladder cancer subtype prediction, none of these modifications resulted in significant improvements (Supplementary Methods). We examined the potential benefit of expanding the pool of available rules by allowing selected differentially expressed genes to form gene-pairs also with the remaining genes in the training data. A non-informative gene may form a highly informative rule by acting as a pivot point for the differentially expressed ones. The classifiers using pivot genes had similar performance to those only using differentially expressed genes and had similar levels of platform specific rules (Supplementary Table S1, Supplementary Fig. S5).

#### 3.1.4 Rule-based Random Forest

**Fig. 3.**
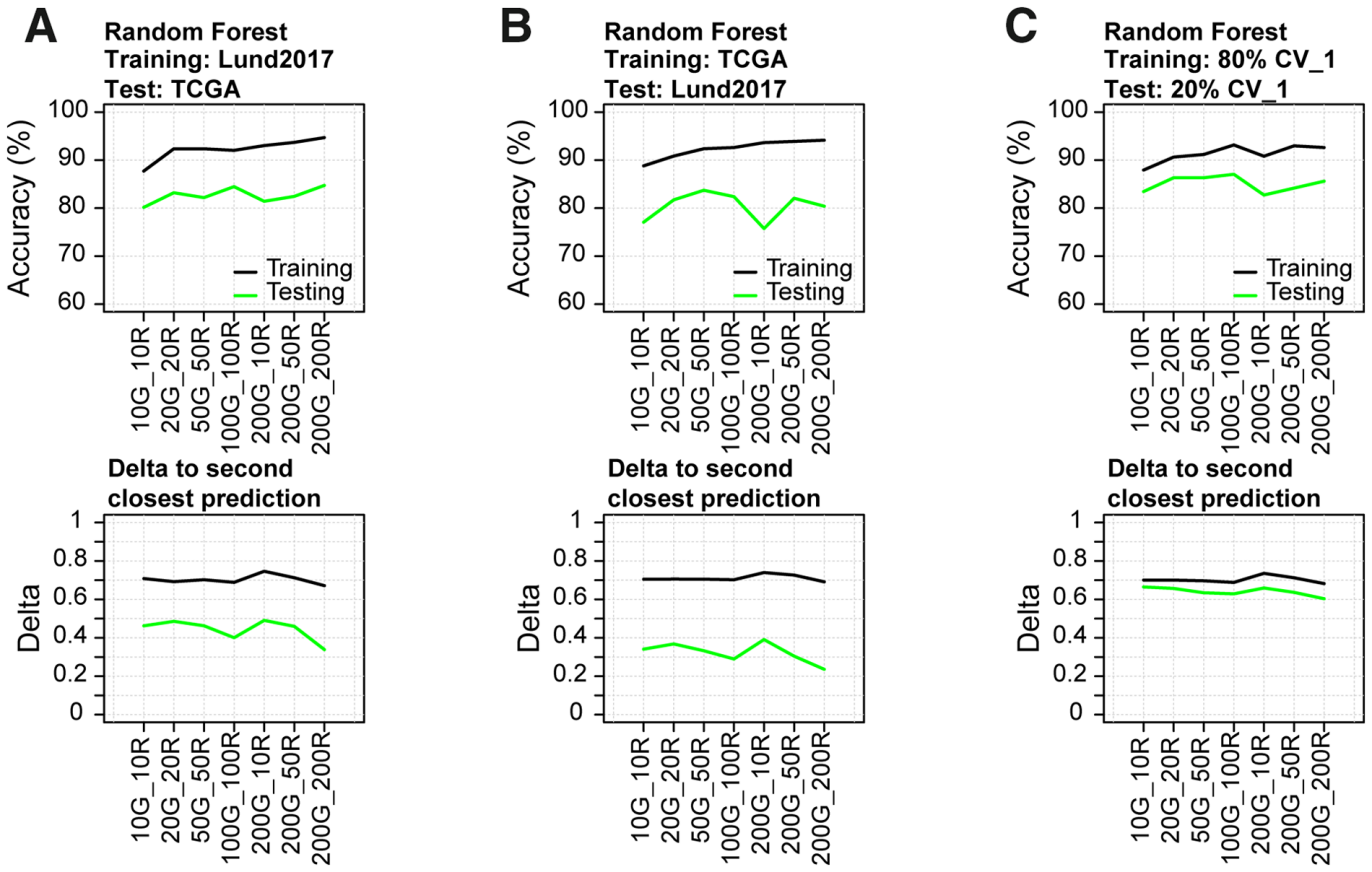
Random Forest model performance. Top row: Training and prediction accuracy across models trained on (**A**) Lund2017, (**B**) TCGA and (**C**) mixed Lund2017/TCGA data Bottom row: Average score difference between the predicted and second closest class

We evaluated seven models with different combinations of input genes (g) and rules (r) per class (10g/10r, 20g/20r, 50g/50r, 100g/100r, 200g/10r, 200g/50r and 200g/200r). The Lund2017 classifiers had OOB accuracies between 0.88 and 0.95 and prediction accuracies between 0.80 and 0.85 applied on TCGA data (Fig.  3A). The TCGA classifiers had OOB accuracies between 0.89 and 0.94, while the accuracy on the Lund2017 dataset ranged between 0.75 and 0.84 (Fig.  3B). Models trained on mixed data had an average OOB accuracy of 0.91 (0.87–0.94) and prediction accuracies between 0.83 and 0.92 on the five test splits (Fig.  3C, Supplementary Table S1). Despite minor differences in prediction accuracy, prediction scores were very consistent between smaller and larger models, with prediction shifts occurring mainly in samples with low prediction scores regardless of model size. In addition, no tied scores were observed even when few genes and rules were utilized. As the RF models use a preset number of rules, we examined if all were informative to the final model. We applied the Boruta algorithm which compares the importance of each feature in the model with permuted versions of themselves. Nearly all rules were considered informative in the smaller models, but in the larger models (100genes/100rules and 200genes/200rules) approximately 30% and 50% of rules were considered uninformative. These could be discarded without any loss in performance (Supplementary Table S1), representing a reduction of required genes by 15% and 30%, respectively. The platform specificity of the RF rules was similar to that observed for AIMS and k-TSP models (Supplementary Fig. S5).

#### 3.1.5 Biological evaluation of predictor output scores

**Fig. 4.**
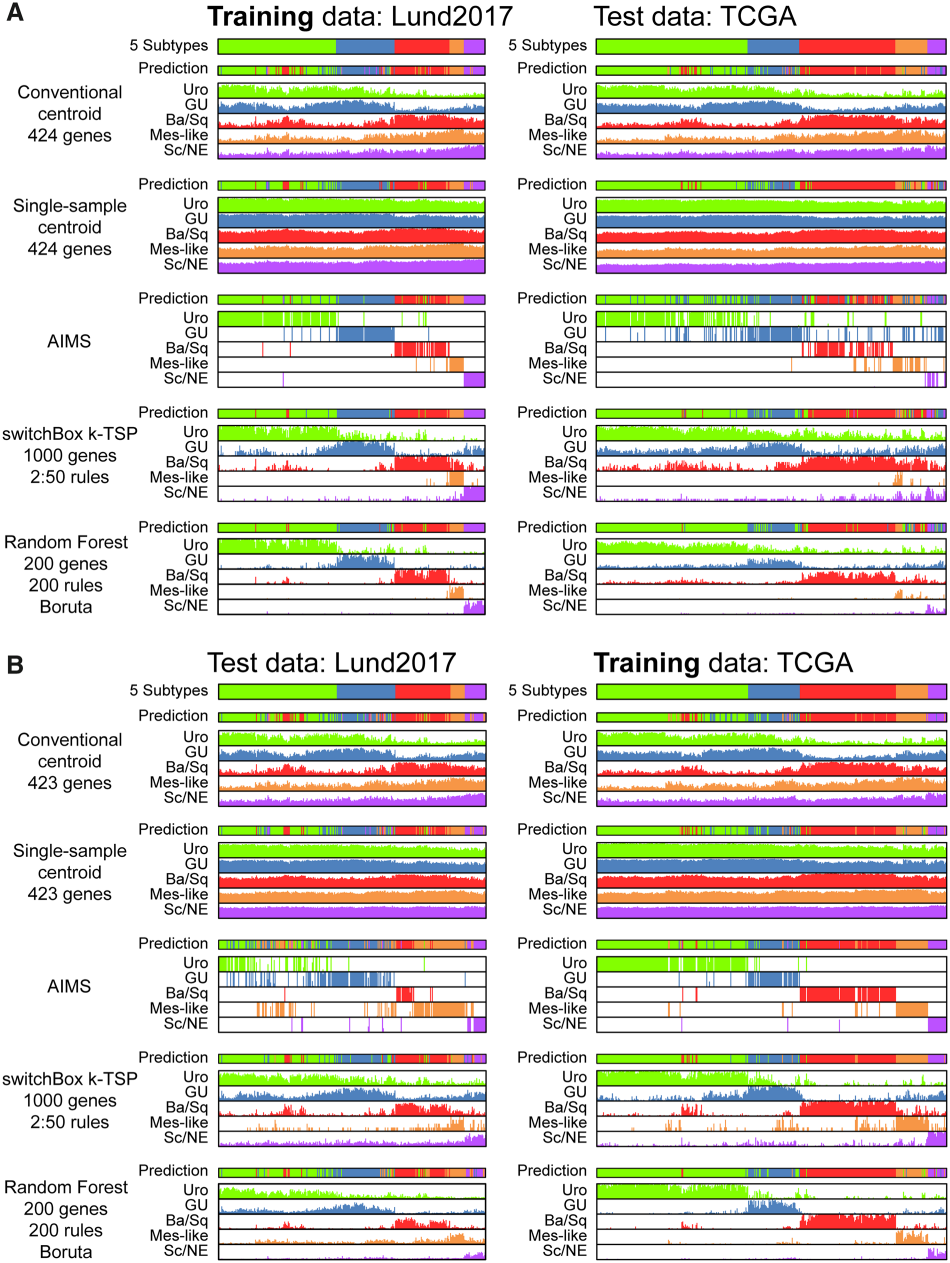
Predictor output scores on bladder cancer data. (**A**) Models trained on Lund2017 data (training predictions left) and applied to the TCGA dataset (right). (**B**) Models trained on TCGA data (training predictions right) and applied to the Lund2017 dataset (left)

Next, we examined the prediction output scores of the different classifiers (Fig.  4). While the accuracy of a classifier is a primary performance metric, the ability to gain additional information from the predictor should also be considered. For the models trained on isolated datasets, we plotted the prediction scores from the training data and the respective test dataset. The subtype calls were, with a few exceptions, similar between the different predictor methods, but with large differences in score behavior. The score (correlation to centroids) for the conventional centroid classifier trained on Lund2017 and TCGA data showed an expected behavior, with a separation between samples with high correlation to the Uro and GU centroids and those with a correlation to the Ba/Sq, Mes-like and Sc/NE centroids, reflecting a biological split between subtypes that retain degrees of urothelial differentiation and those that display divergent differentiation. An expected increased correlation to the Ba/Sq centroid was observed for the UroB subcategory, as both groups have high expression of basal keratins and basal markers. Sample with higher stroma and immune content in the biopsy across the dataset showed increased correlation to the Mes-like centroid. Mes-like tumor cells express mesenchymal genes and downregulate many epithelial genes. Expression profiles from high stroma content biopsies can therefore resemble the Mes-like subtype when compared against less infiltrated cases. Nearly all Ba/Sq and Mes-like tumors have a high infiltration while this is only the case for some Uro and GU tumors (Supplementary Fig. S7). The SS-centroid predictions were similar to the conventional centroid but with diminished differences in correlation between any subtype call, while remaining similarly affected by tumor purity and differentiation state. The AIMS method had the most distinct prediction scores, approximating 1 for the called class and 0 for the remaining classes in most samples, in both training and test data. High prediction scores were however also seen for clear misclassification events, such as predicting any stroma-rich biopsy as Mes-like when the TCGA model was applied to the Lund2017 cohort. The non-informative score leaves uncertain or potentially incorrect predictions harder to identify and interpret. The scores from the k-TSP and RF classifiers were more distinct than the centroids, while more clearly reflecting prediction confidence than the AIMS score. The degree of infiltration did not strongly impact the predictions scores. Notably, both the k-TSP and RF classifiers trained on Lund2017 data indicated that only a smaller number of TCGA samples belonged to the Mes-like class. These samples also had higher Out-of-Bag Mes-like scores in the TCGA model. The Mes-like subtype is difficult to call without proper immunostainings, and hence there is a higher risk of incorrect reference labels in the TCGA dataset. The higher Mes-like recall of the Lund2017 centroids could be interpreted as a drawback of the model if this is achieved by predicting both tumors truly of the Mesenchymal-like subtype as well as those with the highest level of stroma infiltration as Mes-like. Examination of the expression patterns of epithelial and stromal genes across the Mes-like tumors did lend support to the narrower calls made by the k-TSP and RF predictors (Supplementary Fig. S8). The Lund2017 classifiers and TCGA OOB scores also indicated a smaller fraction of TCGA tumors to be of the Sc/NE subtype. The samples most consistently classified as Sc/NE were those that showed the strongest upregulation of neuroendocrine genes (*e.g.,* *TUBB2B*, *CHGA*, *ENO2*, *SYP*), and low expression of many epithelial genes. Notably, the OOB prediction scores of an RF model trained on the full combined dataset closely matched the OOB patterns of the individual dataset models, indicating that the score reflects true biological differences (Supplementary Fig. S9). Given that the prediction scores of k-TSP and RF best reflected known nuances of the data in terms of biological properties and reference label uncertainties and were applicable also to the full 7-class Lund stratification (dividing Urothelial-like tumors into subcategories UroA, UroB and UroC), and to classification according to consensus labels across 18 datasets (Supplementary Methods), we evaluated these two methods further.

### 3.2 Results on additional datasets

#### 3.2.1 Breast cancer

**Fig. 5.**
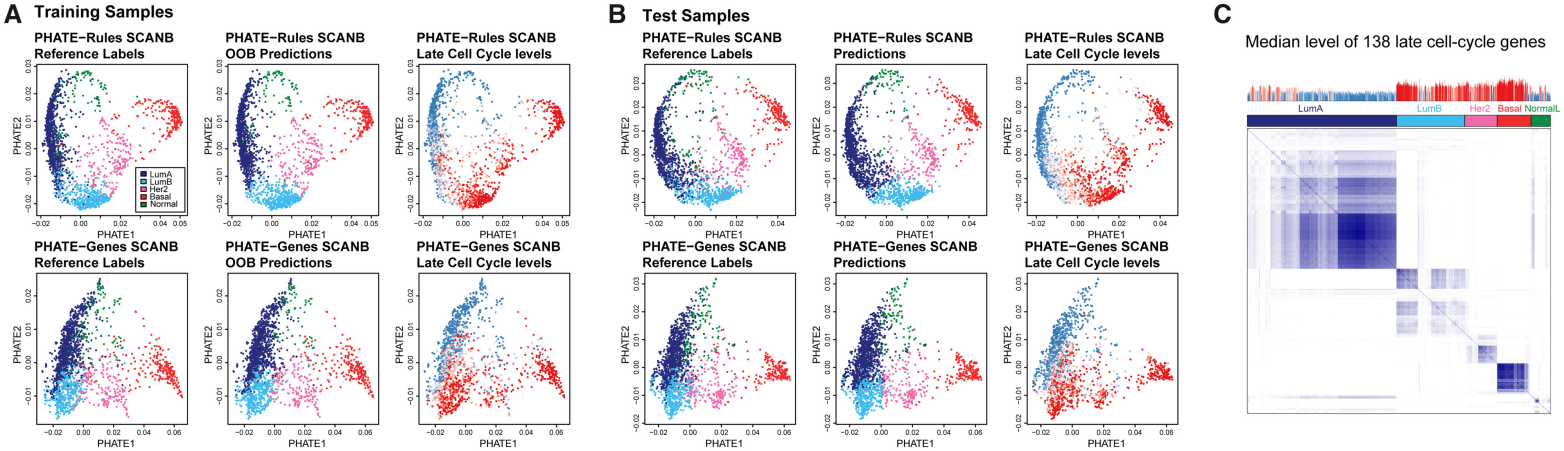
Examination of the RF_200_200_Boruta SCANB breast cancer model. PHATE embedding of classifier rules (top row) and genes (bottom row) of (**A**) training samples and (**B**) test samples, with reference labels, OOB-predictions/predictions and median late cell cycle expression indicated. (**C**) Clustered proximity matrix extracted from the RF classifier, with corresponding per-sample median late cell cycle expression

We used 3814 RNA-sequenced samples from the Swedish Cancerome Analysis Network-Breast project (Brueffer *et al.*, 2018). The k-TPS and RF models were trained to predict Luminal A, Luminal B, HER2-enriched, Basal-like and Normal-like classes on 50% of the samples (*n* = 1907) using supplied centroid-derived reference labels. The k-TSP models had training accuracies between 0.81 and 0.88 and prediction accuracies between 0.78 and 0.85, while RF models had between 0.88 and 0.92 OOB accuracy and 0.86–0.89 prediction accuracy (Supplementary Table S1). To examine the prediction results in more detail, we applied dimensionality reduction on both the rules and genes used by the RF model using PHATE (Moon *et al.*, 2019). For the binary classifier rules, a matrix of precomputed Hamming distance was used as input. Both genes and rules gave similarly coherent representations of the dataset structure (Fig.  5A and B). Samples that received prediction labels not conforming with the reference were either in close proximity to the predicted subtype or in the transitional zone between subtypes. Prediction differences between models were confined to such samples. Breast cancer represents an interesting classification challenge as the transition between Luminal A (LumA) and Luminal B (LumB) is gradual, reflecting a split based on proliferation which is a continuous variable. We noted that proliferation, as measured by the mean log2 expression of 138 late cell-cycle genes, did not conform fully with the reference split between LumA and LumB (Fig.  5). Inconsistencies regarding proliferative markers between breast cancer datasets classified through different methods have been reported (Prat and Parker, 2020), as has concordance issues between different predictor models (Sontrop *et al.*, 2016) and prediction shifts related to cohort centering (Cascianelli *et al.*, 2020; Paquet and Hallett, 2015). More gradual class splits such as LumA/LumB likely warrants careful classifier tuning to achieve reliable cross-dataset performance. Through the RF method, a proximity matrix can be extracted based on the number of times different Out-of-Bag samples end up in the same terminal node during the classifier training (Fig.  5C). This can give an indication of reference class cohesiveness, how broadly inclusive the decision trees are for different classes, and which samples have uncertain or potentially incorrect reference label. In the SCANB dataset, this indicated that a proportion of LumA and B tumors showed lower cohesion with other samples, which largely corresponded with proliferation estimates that deviated from that of their respective subtype (Fig.  5C).

#### 3.2.2 Lung cancer

Next, we evaluated the k-TSP and RF approach in a lung cancer meta-cohort previously utilized to examine SSPs across heterogeneous datasets (Cirenajwis *et al.*, 2020). A set of 1918 samples was used to evaluate of tumor histology prediction (adenocarcinoma (AC) versus squamous cell carcinoma (SqCC)). The models were trained on 1150 samples from seven datasets and applied to a test set of 768 samples from five datasets (Supplementary Table S1). For this binary problem, the k-TSP method outperformed the RF method, with each k-TSP variation selecting between 9 and 13 rules, with a high degree of rule overlap between models and prediction accuracies between 0.91 and 0.92. The RF accuracy varied between 0.69 and 0.92, with low accuracy in two models due to poor accuracy in a large dataset using the older HG U95Av2 Affymetrix platform. Rule behavior in this dataset deviated from the other platforms, which resulted in mixed prediction scores across all k-TSP and RF models but extensive misclassification in two RF models (Supplementary Fig. S10). When classifiers were trained on mixed samples (50%) from all platforms, prediction accuracies on the remaining samples ranged between 0.92 and 0.95 across models (Supplementary Table S1). Subtype prediction among AC tumors into either terminal respiratory unit (TRU) or non-TRU subtype was performed on 2106 samples from 13 datasets, using 1429 training samples (7 datasets) and 677 test samples (6 datasets). Accuracies for the k-TSP models ranged from 0.71 to 0.73, and RF between 0.69 and 0.81, with the same HG U95Av2 Affymetrix dataset being the primary source of misclassification events. Training on mixed samples (50%) from all platforms, the k-TSP achieved 0.86–0.87 accuracy on the remaining samples, while the RF accuracy ranged between 0.85 and 0.92 (Supplementary Table S1).

#### 3.2.3 Pan-cancer

Finally, we applied the rule-based predictor methods on 10 088 samples from the TCGA Pan-Cancer dataset to predict the tumor type, representing a problem with a high number of biologically distinct classes. We used 5044 samples spanning 32 cancer types for training (colon adenocarcinoma (COAD) and rectal adenocarcinoma (READ) merged into a COADREAD class) and performed the prediction on the remaining 5044 samples. The k-TSP methods achieved prediction accuracies between 0.89 and 0.92, while the RF approach (using 25 genes and rules per class and 200 from the ‘All’ model) had an OOB training accuracy of 0.97 and a prediction accuracy of 0.96 (Supplementary Fig. S11, Supplementary Table S1). We observed tied scores for 17–574 cases depending on which k-TSP variation was used, while no scores were tied with the RF method (Supplementary Table S1).

## 4 Discussion

The aim of molecular subtyping is to resolve cancer into homogeneous subgroups based on shared molecular features. Gene expression-based stratification of cancer into subtypes have been explored in most forms of cancer and have demonstrated both prognostic and predictive value. Molecular subtypes are often defined within investigations of relatively small cohorts of dozens to a few hundred tumor samples. To validate a molecular stratification and expand on clinical and biological associations, a common approach is to develop a prediction model that can be applied to new samples or publicly available datasets. Commonly used classification methods utilized in research are often trained on, and applied to, row-centered gene expression data (genes in rows and samples in columns), meaning that they rely on relative expression differences between samples. Prediction results from such classifiers can be severely affected if the subtype distribution of a new cohort differs significantly from that of the training cohort. If the data from the new cohort has been generated with the exact same method it is possible to effectively normalize and integrate it for compliance with a prediction model. However, as research cohorts from different studies can be generated on diverse platforms and may differ in many other clinical, biological or methodological aspects, such normalization strategies are often challenging. A SSP is applied to a new sample in isolation without relying on cohort row-centering, effectively bypassing the issue of relative expression differences. This approach is advantageous in *e.g.,* clinical tests or prospective molecular subtyping, where invariant predictions of individual samples are needed. Similar to relative classifiers, this is simplified if the platform remains identical. Given the large amount of publicly available transcriptomic research data across diverse platforms, a SSP with robust performance also across platforms would be highly useful. The rule-based classifiers that were trained on isolated bladder cancer datasets showed transferability between array and RNA-seq platforms, performed well when trained on two bladder cancer datasets and applied to 16 separate cohorts or vice versa, and performed well on most dataset in lung cancer. In the lung cancer evaluation, we saw platform issues with an older microarray dataset that was not represented in the training cohort. The rule-based classification approach boils down to the identification and utilization of subtype-informative gene-pair ratios that are captured across gene expression profiling methodologies. Gene-length normalized RNA-sequencing data (*e.g.,* TPM or FPKM) can be expected to better reflect the quantitative ranking of gene mRNAs compared with signal intensities from microarrays (Smid *et al.*, 2018). Correlations ranging roughly between 0.6 and 0.8 have been reported between RNA-sequencing and microarray signal intensities (Black *et al.*, 2014), which may vary further depending on platform and data preprocessing approach (*e.g.,* RMA, GCCN and SST). If data from a new platform deviates greatly in dynamic range from that of the training cohort, or does not at least partly reflect true mRNA quantities, a number of rules may be affected to the detriment of the prediction results. Therefore, as with any research classifier, compatibility with new data should be evaluated and verified, particularly if the expression platform was not represented in the training data. Inclusion of mixed cohorts during training improved the cross-platform behavior of the selected rules, and this approach has been used in published rule-based classifiers such as the AIMS breast cancer subtype predictor. This could potentially be extended further using additional versions of the training data, preprocessed using different RNA-seq quantification or microarray summarizing methods to find stable rules. We found that the rule-based SSPs performed excellent when trained and tested on 50/50 data-splits of SCANB and Pan-Cancer TCGA data, indicating robust performance when applied to uniformly generated data. Prediction results in most tests were also stable even when relatively few genes were used, suggesting that these methods may be applicable to panel-based mRNA quantification methods like Nanostring nCounter. Many tumor classification systems include sample categories partly defined by their high degree of non-tumor cells. Such infiltrated categories can be relatively uninformative regarding tumor cell features. Intrinsic tumor cell properties certainly play a role in the composition of the tumor mass, *e.g.,* by being immune-attracting or having a propensity for invasive growth, but low purity can also be caused by general inflammation or tissue sampling. Bladder cancer tissue specimens may come from papillary (exophytic), flat or inverted (endophytic) or invasive lesions, contributing to the variability of biopsy cellularity even if tumor-rich areas are microdissected for analysis. An analysis of purity across TCGA cohorts indicated that bladder cancer had among the lowest average purity scores and high variability between samples (Aran *et al.*, 2015). Tumor purity can affect transcriptomic data interpretation both during subtype discovery and classification of new samples. As gene expression preprocessing often involves normalization steps where data distributions are matched across samples, *e.g.,* through quantile normalization, expression originating strictly from tumor cells will appear lower in low purity tumor biopsies. This affects both clustering and many prediction models. Our previous studies of bladder cancer, employing both gene expression and immunohistochemistry, revealed that discrete tumor cell phenotypes determined by IHC often drift apart during gene expression clustering due to differences in purity (Sjödahl *et al.*, 2017). The rule-based predictors relative invariance to purity suggests that they could be useful in classification tasks where cellularity poses a problem. As immunological and stromal properties of the tumor can also be biologically and clinically important, a more comprehensive approach could entail calling the molecular subtype of the tumor cells, and separately assessing purity, immune and microenvironment features. Rule-based SSPs have shown applicability also in these latter tasks. For example, prediction of tumor immune properties has been demonstrated across TCGA cohorts (Gibbs, 2020), and expression-level estimations of various gene pathways have been demonstrated in breast cancer (Paquet *et al.*, 2017). Rule-based methods may prove valuable in many research efforts, such as consolidating transcriptomic profiling results from multiple studies, performing prospective tumor subtyping, evaluating existing classifications, and building preprocessing-free gene-panel-based predictors. The k-TSP and the new RF method most closely matched the properties we sought in a multiclass single-sample classifier and have been made available through our R package ‘multiclassPairs’ which allows for easy training and prediction as well as extensive parameter tuning by the user.

## Supplementary Material

btab763_supplementary_data
