# Supplementary material for: A comparison of rule-based and centroid single-sample multiclass predictors for transcriptomic classification
Source: Bioinformatics. 2021 Nov 12;38(4):1022–9. doi: 10.1093/bioinformatics/btab763 (PMC8796360; doi:10.1093/bioinformatics/btab763)
Supplement: btab763_supplementary_data [file btab763_supplementary_data.zip › BioinformaticsSupplement.docx]

**Supplementary Information**

**switchBox k-TSP variations**

Several variations to the default gene filtering and rule-scoring approach of switchBox were examined. Overall, none of these approaches resulted in notable uniform improvements in accuracy towards the reference labels in the bladder, lung, or breast cancer datasets (Table S1). The stability of peak accuracy and concordance between tests, both using k-TSP variations and RF models, suggests that there may be a limit to the obtainable accuracy. While this could be interpreted as a shortcoming of the classifier methods, it is more likely caused by reference label uncertainty, tumor heterogeneity, or deviating tumor biology.

**One-vs-one gene filtering.** The switchBox k-TSP method is designed with binary classification in mind, and the initial gene filtering step is therefore performed by 1-vs-rest testing. We examined whether performing gene filtering through 1-vs-1 testing resulted in performance increases. This was done through a Kruskal–Wallis test followed by a Dunn’s test to select genes that were differently expressed against all other classes. This extension did not show significant improvement.

**Platform-wise gene filtering.** When the training data is composed of different datasets/platform platform-wise filtering step could be considered, especially when non-normalized data is used. In this case the gene filtering step is performed separately for each platform, and the overlap of significant genes across the platforms are used to generate the rules. We performed platform-wise gene filtering retaining the top genes in all platforms/studies. This extension did not show significant improvement.

**Platform-wise rule scoring**. An option for multi-platform training is to calculate the scores for the rules separately for each platform and then average their scores. We applied this principle to the mixed datasets by selecting genes with a platform-wise 1-vs-1 filtering and then calculated the rule-scores as the average from each platform. This extension did not show significant improvement.

**One-vs-one rule scoring**. By default, the score of a rule is calculated as class-vs-rest, as the percentage of this rule (i.e., when it is true) in the class minus the percentage of this rule in other classes together. We modified this score to take each class in account separately. We calculated the score against each individual class separately and averaged the scores. This extension did not show significant improvement.

**Lund Taxonomy 7-class prediction**

We employed the k-TSP and RF methods for the full 7-class Lund stratification where Urothelial-like tumors are substratified into UroA, UroB, and UroC. The same issue of tumor purity applies, but the prediction task becomes more challenging as UroB shares some features with the Ba/Sq subtype while UroC shares some features with the GU subtype (Bernardo et al., 2019; Sjödahl et al., 2017). The k-TSP method struggled to identify high numbers of rules unique to each class. With fewer rules, any platform specific rule behavior had a more pronounced effect on the prediction score which resulted in more tied predictions. The overall best performing k-TSP approach was to use a hierarchical classification strategy, first performing the 5-class split and then applying a separate UroA/UroB/UroC k-TSP classifier. The RF method performed better on this task and could be trained to predict all classes at once. For all models, misclassifications were more frequent between datasets, again suffering from platform specific rules, but also from the less precise delineations of this substratification, particularly in the TCGA dataset. Nonetheless, a 7-class RF predictor trained on full mixed Lund2017 and TCGA data and applied to an external dataset of advanced bladder tumors (IMvigor210) (Mariathasan et al., 2018) resulted in a subtype prediction with highly coherent transcriptional profiles and excellent stratification of subtype-associated mutations among these most transcriptionally similar subtypes. Among UroA, UroB, UroC, and GU tumors respectively, FGFR3 mutation were present in 75% (27/36), 40% (8/20), 11.7% (7/60), and 0% (0/39) of tumors, while TP53 was mutated in 16.7%, 20%, 58%, and 89.7%. RB1 was mutated in 5.6%, 5%, 11.7%, and 38.5%, while CDKN2A was deleted in 39%, 15%, 10% and 0% of cases, respectively.

**Consensus subtype 6-class prediction**

The bladder cancer consensus classification project is based on the application of classification systems of different research groups to a range of datasets (Kamoun *et al.,* 2020). From these results, samples with consistent classification results, as evaluated by a weighted network approach, were defined as “core” samples for a new classification consisting of 6 subtypes (luminal papillary (LumP), luminal nonspecified (LumNS), luminal unstable (LumU), stroma-rich, basal/squamous (Ba/Sq), and neuroendocrine-like (NE-like). From the 1084 cases, 403 samples from Lund2017 dataset and the TCGA dataset were used to find significant genes of these subtypes, after which the single sample centroid was defined expression values of the Lund2017 dataset. Following the same approach, we used these 1084 samples, representing 18 datasets of widely different dynamic range and average expression values, to train classifiers on the 403 samples of Lund2017 and TCGA (Partition 1), and applied these to the 681 remaining samples from 16 datasets (Partition 2), as well as in the reversed manner. A Random Forest predictor trained on the 403 samples (Partition 1), had 0.98 OOB accuracy, and a prediction accuracy of 0.93 on the 16 datasets of Partition 2, indicating cross-platform applicability (Table RF 1). Conversely, training the models on the 681 Partition 2 samples followed by application to the 403 Partition 1 samples we obtained 0.98 OOB, and a prediction accuracy of 0.93 (Table RF 2). The k-TSP models had moderately lower overall accuracies around 0.84 when applied to Partition 2, and 0.88 when trained on the 16 datasets and applied to the Partition 1 samples. The small “Luminal nonspecified” group, bearing this name as no clear distinguishing features were identified, was the only class with poor prediction performance. This class showed mixed agreement between the contributing classification results defining the group in the original publication (Figure 1 in Kamoun *et al.,* 2020). At this stage, it cannot be concluded if the absence of cases classified as LumNS is caused by limitations of the classifier or is a consequence of a poorly defined subtype.


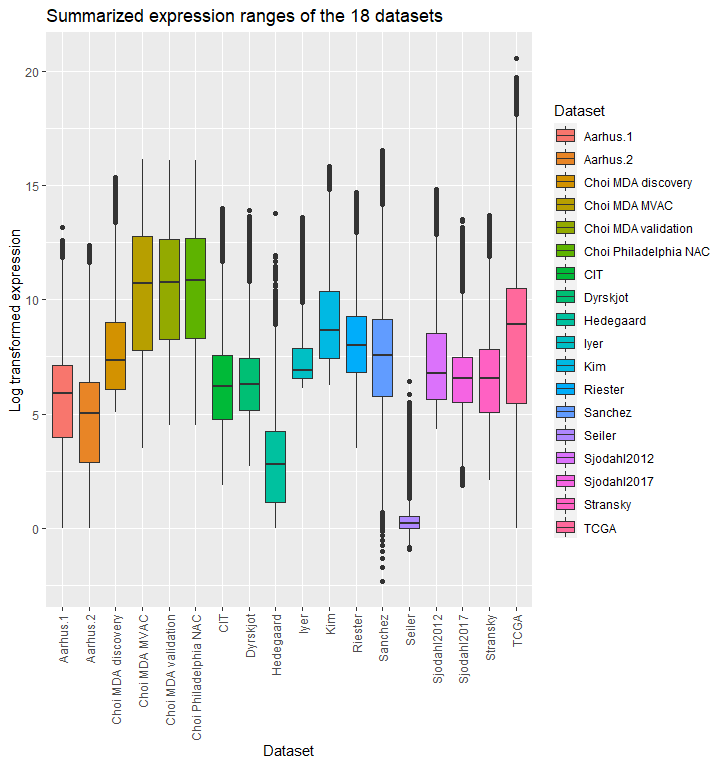


Random Forest 200_200 model confusion matrix

Table RF 1

Train on Partition 1 (n=403), Test on Partition 2 (n=681)

Confusion Matrix and Statistics

Reference

Prediction LumP LumNS LumU Ba/Sq NE-like Stroma-rich

LumP 113 2 12 1 0 0

LumNS 0 0 0 0 0 0

LumU 0 7 98 0 5 2

Ba/Sq 0 0 1 302 4 0

NE-like 0 0 0 0 12 0

Stroma-rich 0 2 5 3 2 110

Table RF 2

Train on Partition 2 (n=681), Test on Partition 1 (n=403)

Confusion Matrix and Statistics

Reference

Prediction LumP LumNS LumU Ba/Sq NE-like Stroma-rich

LumP 95 6 4 0 0 0

LumNS 0 0 0 0 0 0

LumU 1 4 54 0 0 1

Ba/Sq 1 0 1 171 0 5

NE-like 0 0 0 0 8 0

Stroma-rich 0 4 1 0 0 47

Overall accuracy and balanced accuracy of k-TSP and RF models applied to mixed datasets of the consensus cohort

|  | **Train on Partition 1 (n=403), Test on Partition 2 (n=681)** | | Overall Accuracy | | LumP | LumNS | LumU | Ba/Sq | NE-like | Stroma-rich |
| --- | --- | --- | --- | --- | --- | --- | --- | --- | --- | --- |
| k-TSP | Default (Up to 10) |  | 0.8414 |  | 0.8798 | 0.583446 | 0.8698 | 0.9468 | 0.78261 | 0.8939 |
| k-TSP | Default (Up to 50) |  | 0.8179 |  | 0.8754 | 0.537246 | 0.8636 | 0.9309 | 0.67391 | 0.9012 |
| k-TSP | 1000 genes |  | 0.8414 |  | 0.917 | 0.585685 | 0.8699 | 0.9378 | 0.630435 | 0.918 |
| k-TSP | 1000 genes - Pivot |  | 0.8399 |  | 0.9232 | 0.49478 | 0.8526 | 0.9355 | 0.71739 | 0.9271 |
| k-TSP | 1-vs-1 genes |  | 0.8473 |  | 0.9214 | 0.535007 | 0.8391 | 0.9516 | 0.78261 | 0.9172 |
| k-TSP | 1-vs-1 genes - Pivot |  | 0.8488 |  | 0.9187 | 0.49328 | 0.8638 | 0.9404 | 0.71739 | 0.9405 |
| RF | 10-10 (Boruta) |  | 0.8326 |  | 0.9037 | 0.5 | 0.8407 | 0.9216 | 0.78185 | 0.9299 |
| RF | 20-20 (Boruta) |  | 0.8811 |  | 0.9497 | 0.499254 | 0.872 | 0.9518 | 0.78261 | 0.954 |
| RF | 50-50 (Boruta) |  | 0.906 |  | 0.9745 | 0.545455 | 0.8878 | 0.9679 | 0.76087 | 0.9754 |
| RF | 100-100 (Boruta) |  | 0.9369 |  | 0.9877 | 0.590163 | 0.8972 | 0.9884 | 0.80435 | 0.9877 |
| RF | 200-10 (Boruta) |  | 0.8546 |  | 0.9143 | 0.5 | 0.852 | 0.9448 | 0.630435 | 0.9218 |
| RF | 200-50 (Boruta) |  | 0.9207 |  | 0.9709 | 0.499254 | 0.9029 | 0.9841 | 0.76087 | 0.9635 |
| RF | 200-200 (Boruta) |  | 0.9325 |  | 0.9868 | 0.5 | 0.91 | 0.9868 | 0.76087 | 0.9805 |
|  |  |  |  |  |  |  |  |  |  |  |
|  |  |  |  |  |  |  |  |  |  |  |
|  | **Train on Partition 2 (n=681), Test on Partition 1 (n=403)** | | Overall Accuracy | | LumP | LumNS | LumU | Ba/Sq | NE-like | Stroma-rich |
| k-TSP | Default (Up to 10) |  | 0.8635 |  | 0.8953 | 0.71787 | 0.8767 | 0.9731 | 1 | 0.9008 |
| k-TSP | Default (Up to 50) |  | 0.8734 |  | 0.8747 | 0.95758 | 0.8869 | 0.9789 | 1 | 0.8985 |
| k-TSP | 1000 genes |  | 0.8834 |  | 0.8988 | 0.79187 | 0.889 | 0.9957 | 1 | 0.87593 |
| k-TSP | 1000 genes - Pivot |  | 0.8933 |  | 0.9143 | 0.79444 | 0.9183 | 0.9935 | 1 | 0.85706 |
| k-TSP | 1-vs-1 genes |  | 0.8586 |  | 0.9107 | 0.9383 | 0.77063 | 0.9877 | 1 | 0.8934 |
| k-TSP | 1-vs-1 genes - Pivot |  | 0.8958 |  | 0.9281 | 0.92958 | 0.88 | 0.9935 | 1 | 0.85706 |
| RF | 10-10 (Boruta) |  | 0.8635 |  | 0.9353 | 0.5 | 0.80355 | 0.9741 | 0.625 | 0.9051 |
| RF | 20-20 (Boruta) |  | 0.9156 |  | 0.9696 | 0.5 | 0.9365 | 0.9806 | 0.75 | 0.9282 |
| RF | 50-50 (Boruta) |  | 0.9256 |  | 0.978 | 0.5 | 0.9644 | 0.9763 | 1 | 0.8934 |
| RF | 100-100 (Boruta) |  | 0.9355 |  | 0.9916 | 0.5 | 0.9594 | 0.9849 | 1 | 0.9168 |
| RF | 200-10 (Boruta) |  | 0.8908 |  | 0.9457 | 0.568858 | 0.8771 | 0.9698 | 0.9375 | 0.8914 |
| RF | 200-50 (Boruta) |  | 0.9181 |  | 0.9614 | 0.535714 | 0.9304 | 0.9784 | 1 | 0.9102 |
| RF | 200-200 (Boruta) |  | 0.9305 |  | 0.9734 | 0.5 | 0.9413 | 0.9849 | 1 | 0.9363 |

**Figure S1**

**
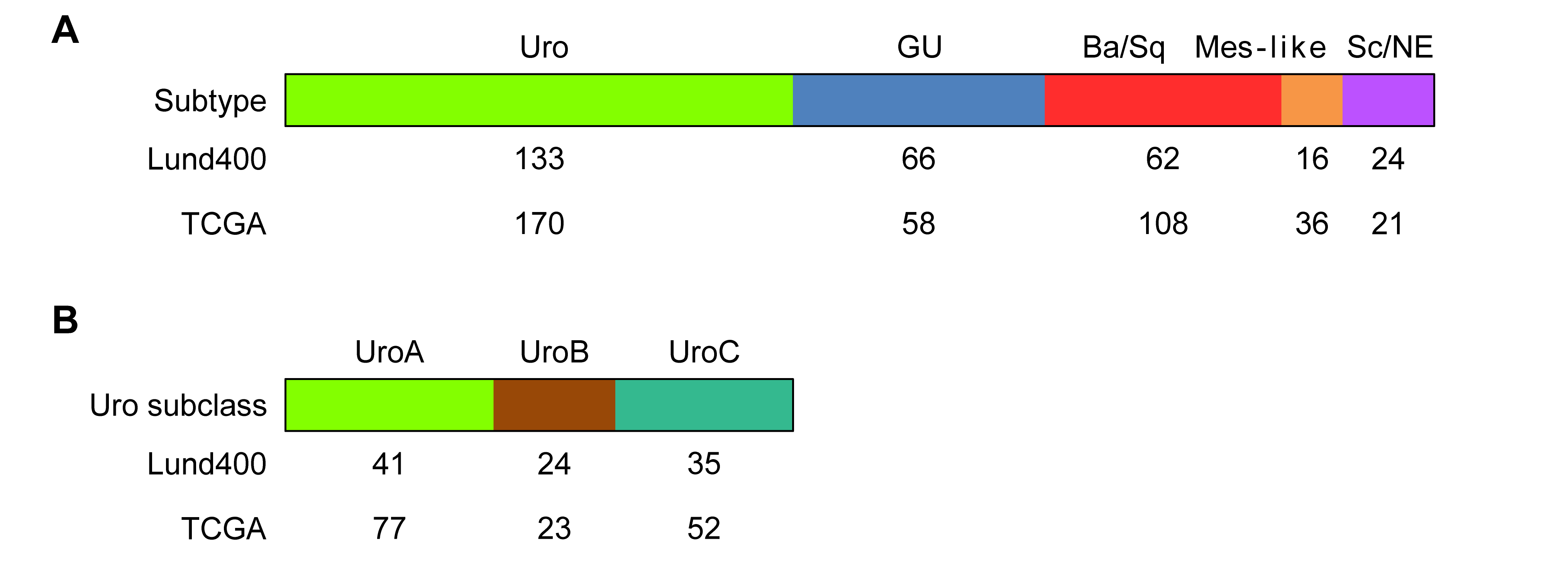
**

**Molecular subtypes across the Lund2017 and TCGA dataset.** A) 5-class subtype division of Lund2017 dataset (Sjödahl et al., 2017) and TCGA-BLCA dataset (Marzouka et al., 2018) into Urothelial-like (Uro), Genomically Unstable (GU), Basal/Squamous-like (Ba/Sq), Mesenchymal-like (Mes-like), and Small cell/Neuroendocrine-like (Sc/NE). B) Sub-stratification of Uro tumors into UroA, UroB, and UroC. Among the Uro samples of Lund2017 and TCGA, 33 and 18 samples respectively could not be confidently sub-classified.

**Figure S2**


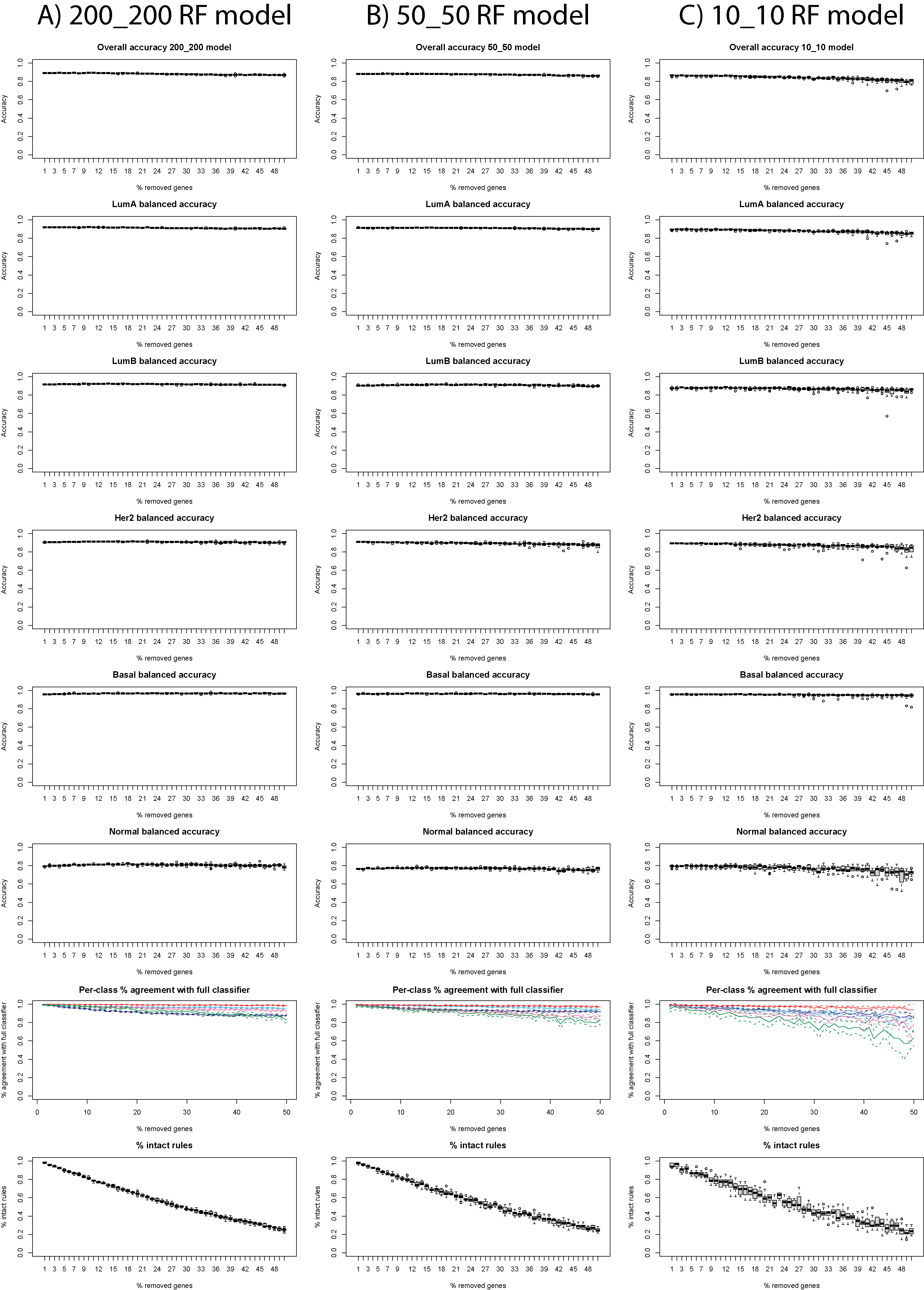


**kNN-based rule-imputation results, illustrated using the SCAN-B breast cancer dataset using 50% of the cohort as test data.** Between 1-50% of genes were removed at random from the test dataset (each repeated 10 times). The rules not able to be generated from the remaining genes were imputed by kNN, after which prediction was performed. Three different Boruta filtered RF models were used: A) 200_200 model B) 50_50 model C) 10_10 model. The upper panels indicate overall accuracy and balanced accuracy towards the reference labels, while the last two panels indicate percent per class agreement with the prediction made when all genes were present, and fraction of intact rules. RF predictions were consistently more accurate than solely calling classes based on the most similar kNN-samples.

**Figure S3**

**
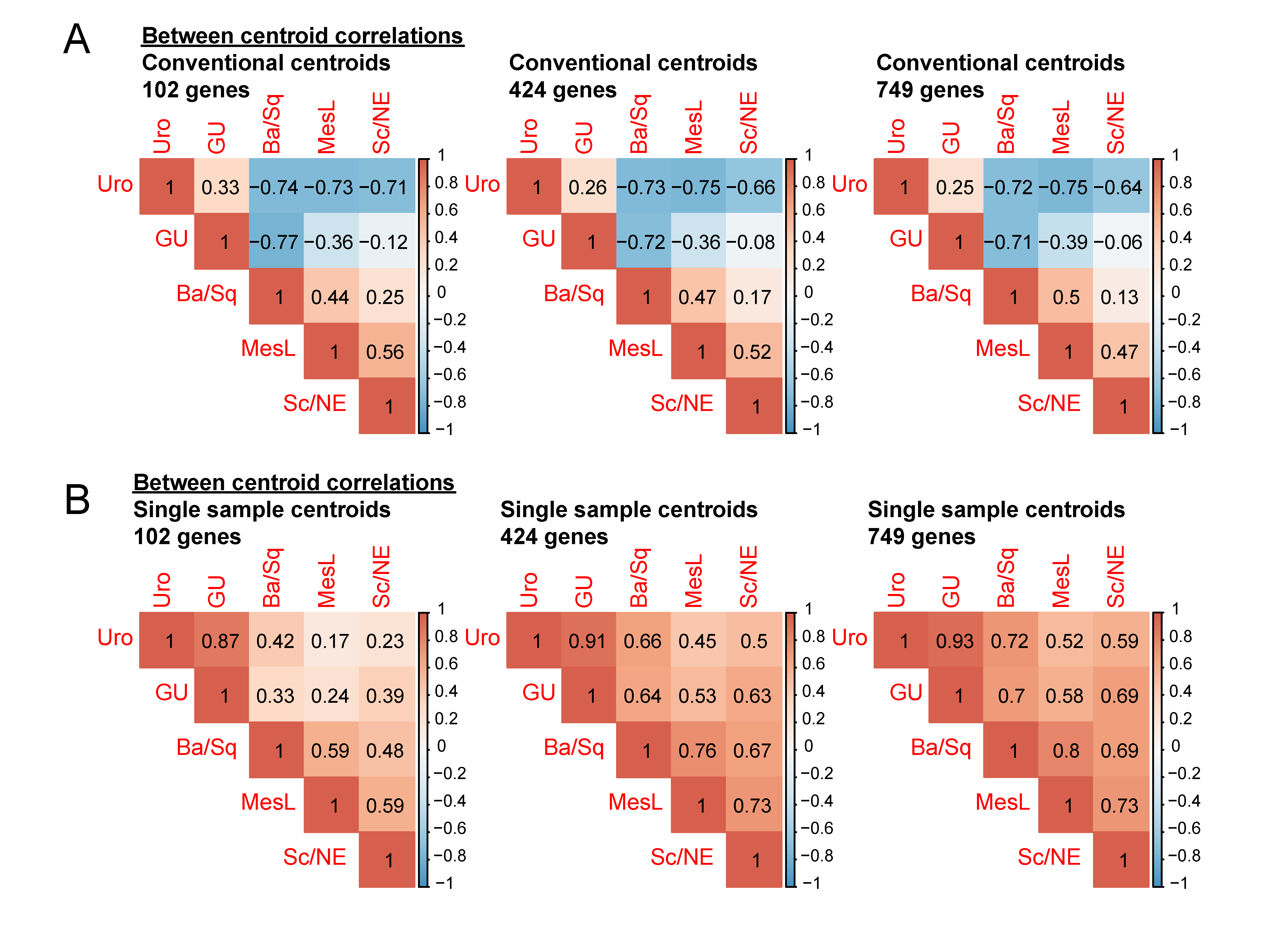
**

**Correlation between classifier centroids.** A) Centered data centroids with 102, 424, or 749 genes, vs B) raw expression SS-centroids with 102, 424, or 749 genes. Correlation between conventional centroids remains comparable, while the correlation between SS-centroids increases.

**Figure S4**

**

**

**AIMS models – platform specific rules.** A visualization of the rules selected and used by the AIMS models. Red arrows indicate gene rules where rules from the training data shows a strongly platform-specific behavior, either being TRUE or FALSE across the majority of the test data. A) Lund 2017 (left) as training data, using TCGA-BLCA as test data. B) TCGA (left) as training data, using Lund2017 as test data. C) 80% of mixed Lund2017/TCGA (CV 1) as training data, with the remaining 20% of samples as test data.

**Figure S5**


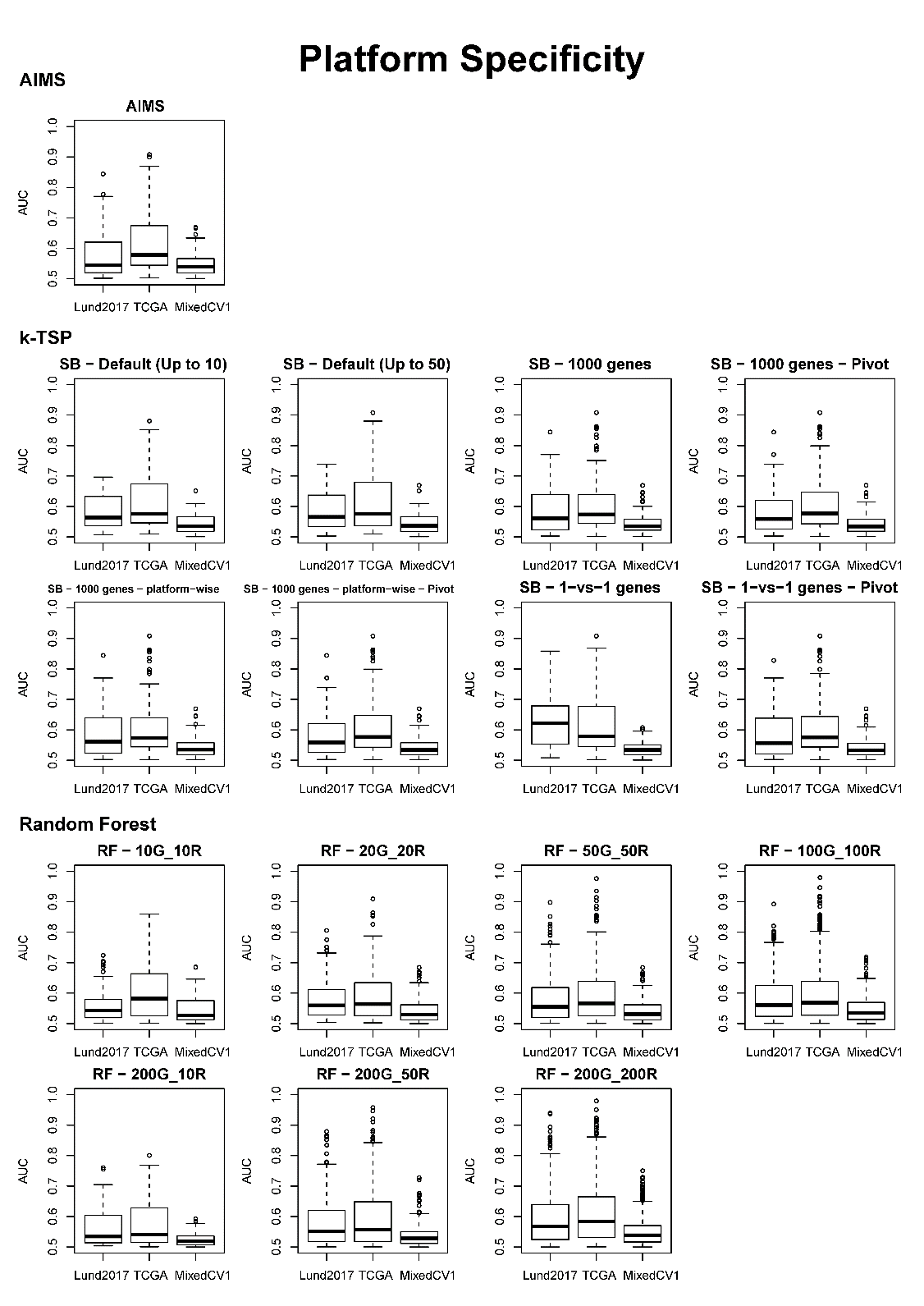


**AUC boxplots of gene pair rules selected by different bladder cancer model variations.** The general trend is an intermediate level of platform specificity across models trained on array data (Lund2017), with an increased level in nearly all models trained on RNA-seq data (TCGA). Each method selected fewer platform specific rules when trained on mixed data.

**Figure S6**

**
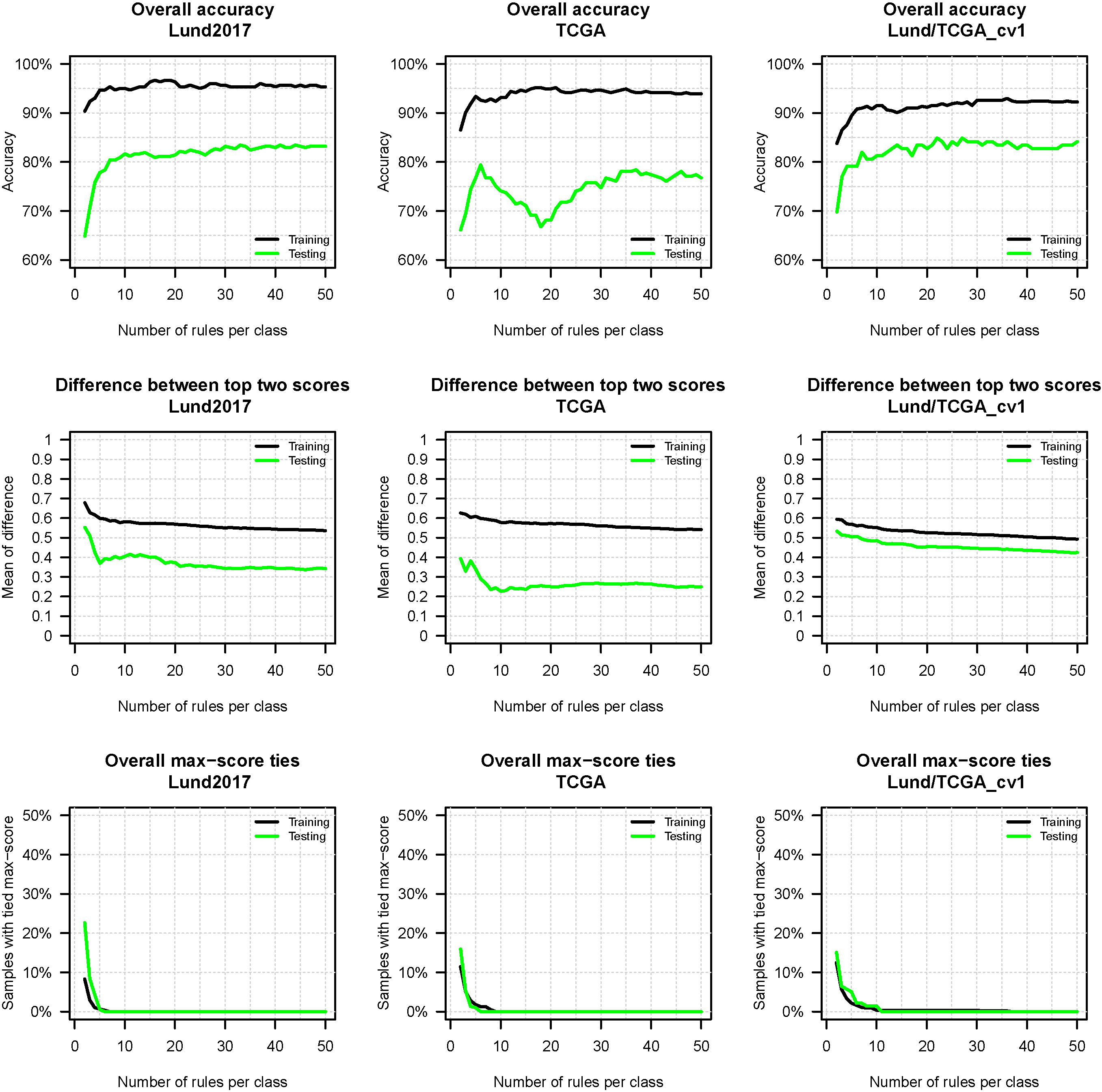
**

**Examination of forcing the number of switchBox k-TSP rules.** The top panels indicate the training and prediction accuracy of models trained on Lund2017, TCGA, or 80% of mixed Lund/TCGA data. The second panel row indicates the change in average difference between the predicted class and second closest prediction. The difference in prediction delta score between the Lund2017, TCGA, and mixed data partly reflects the degree of platform specific rules (Figure S4/S5) that interferes with the prediction clarity. The bottom row indicates the percentage of cases where the prediction score was tied between two or more subtypes.

**Figure S7**

**
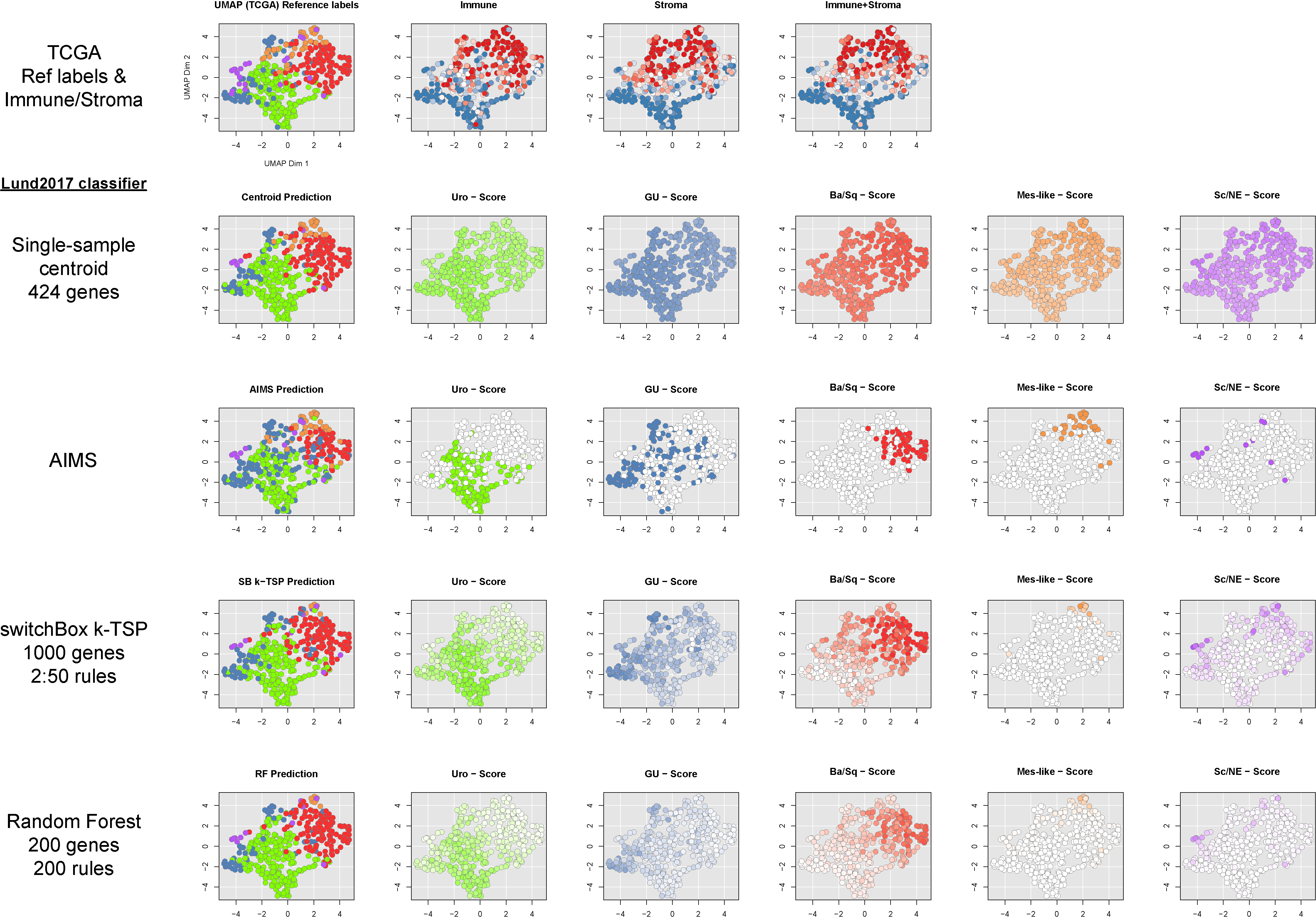
**

**UMAP visualization of TCGA-BLCA dataset.** The top row indicates reference labels of the TCGA dataset as called in Marzouka et al (2017), with average expression of ESTIMATE immune and stroma genes indicated (Yoshihara et al (2013)). Subsequent rows indicate the subtype calls and scores for a SS-centroid, AIMS, k-TSP, and RF model.

**Figure S8**

**

**

**Mes-like tumor prediction in the TCGA TCGA dataset.** The figure shows the Out-of-bag Mes-like scores for the RF 200_200 model trained on the TCGA dataset, and Mes-like prediction scores by the RF 200_200 model trained on Lund2017 data. Downregulated genes in a Mes-vs-Rest SAM analysis highlight a sharp decreased expression of an extensive set of epithelial genes specific to samples with high Mes-like prediction scores. Upregulated genes are a broad mix of immune and stroma related genes which also spike in other infiltrated samples across the TCGA dataset.

**Figure S9**

**
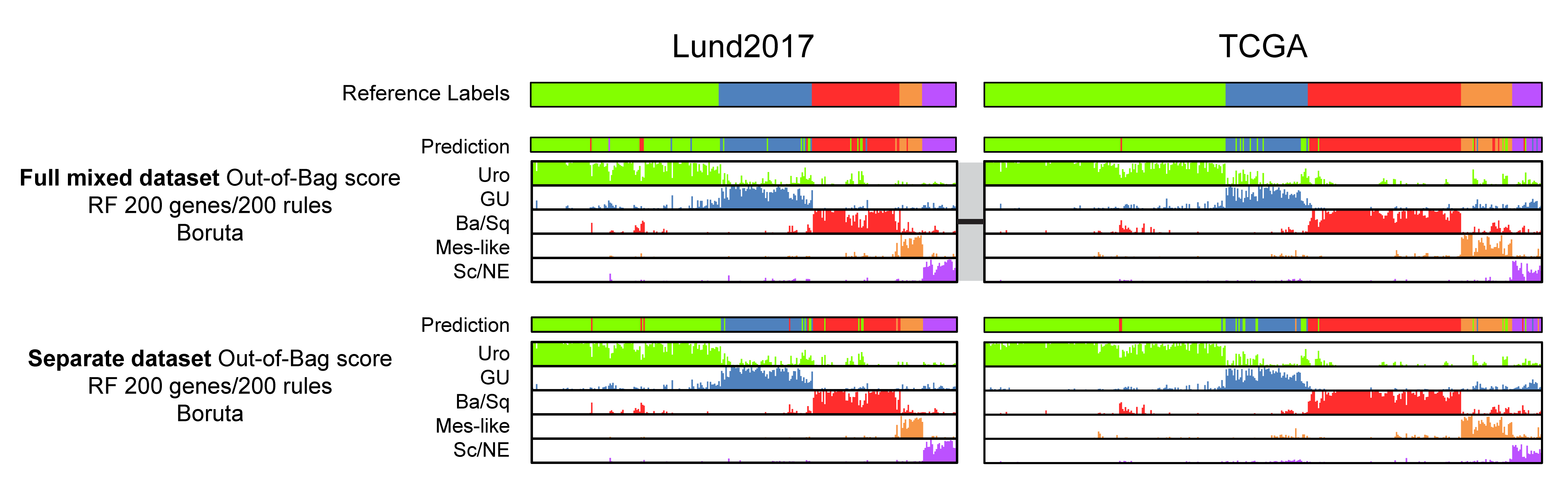
**

**Comparison between RF Out-of-Bag scores.** The OOB scores from a model trained on the full mixed cohort of Lund2017+TCGA (top) closely reflected the OOB scores from the individual models trained on Lund2017 (bottom-left) and TCGA (bottom-right). The scores from all three models accurately reflected uncertainties of the reference labels that we have observed during work on the two cohorts, stemming both from classification challenges (e.g., Mes-like in TCGA), tumor heterogeneity, or subtype similarity.

**Figure S10**

**
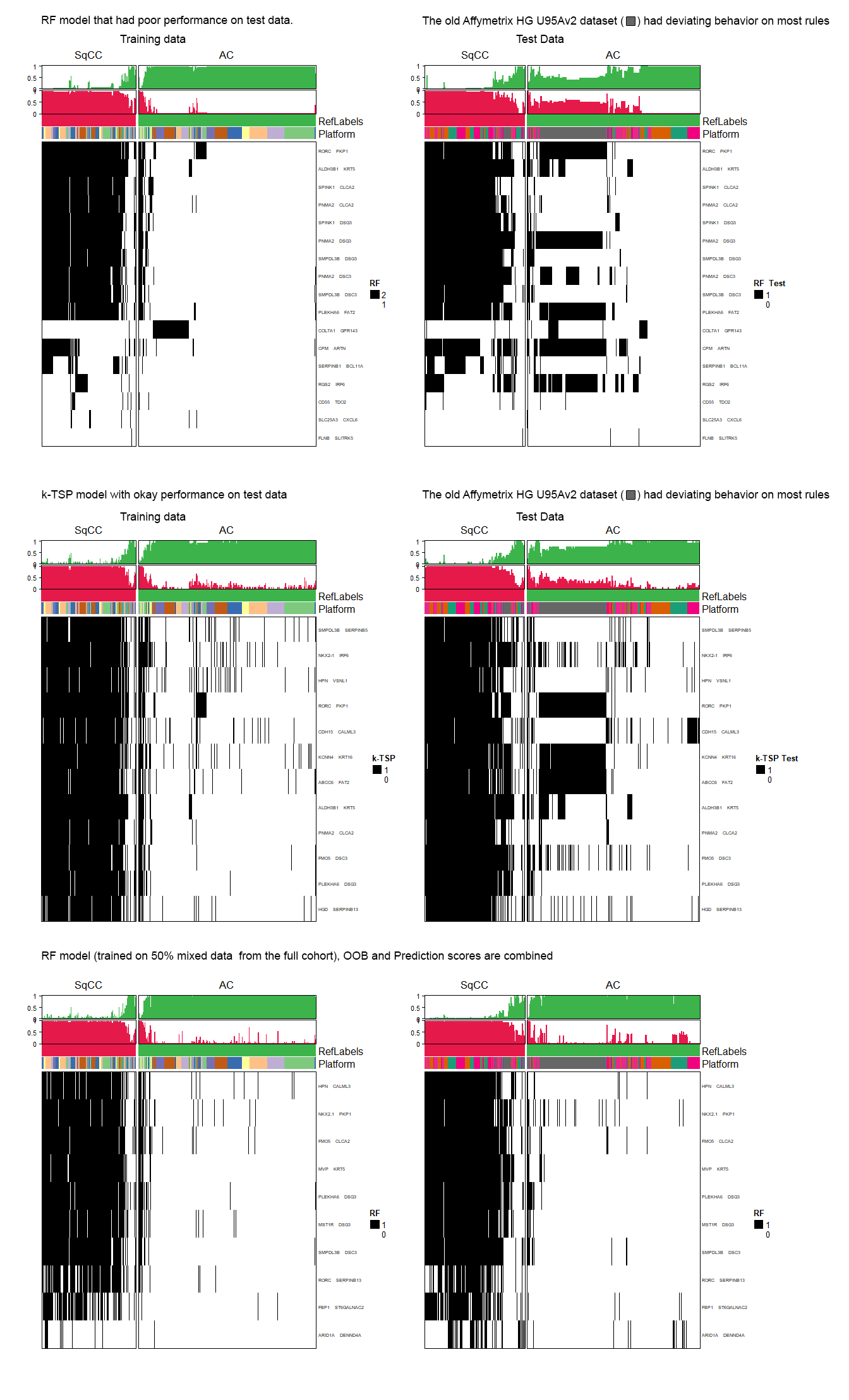
**

**Lung cancer histology classification platform incompatibility.** Here we show one of the small RF models with poor performance on the U95v2, and one k-TSP model. In the figure, the top row indicates the RF model the test samples (right column). Many gene-pairs failed on the older U95 Affy dataset in the test set (indicated in gray, right column). The second row has the samples in the exact same order, showing the 1000 genes k-TSP model, which had better accuracy, but similar trend on scores, both on the U95 dataset and on other “misclassifications” (right side of SqCC, and left side of AC). The consistent call of these samples (across all models) may indicate an incorrect reference label, or tumor heterogeneity. The bottom row illustrates a small RF model trained on 50% randomly selected samples from the entire cohort. The combined OOB and prediction scores, matched in order, recaptures the “misclassification events”, but does not have an issue on the Bhattacharjee et. al U95 dataset.

**Figure S11**


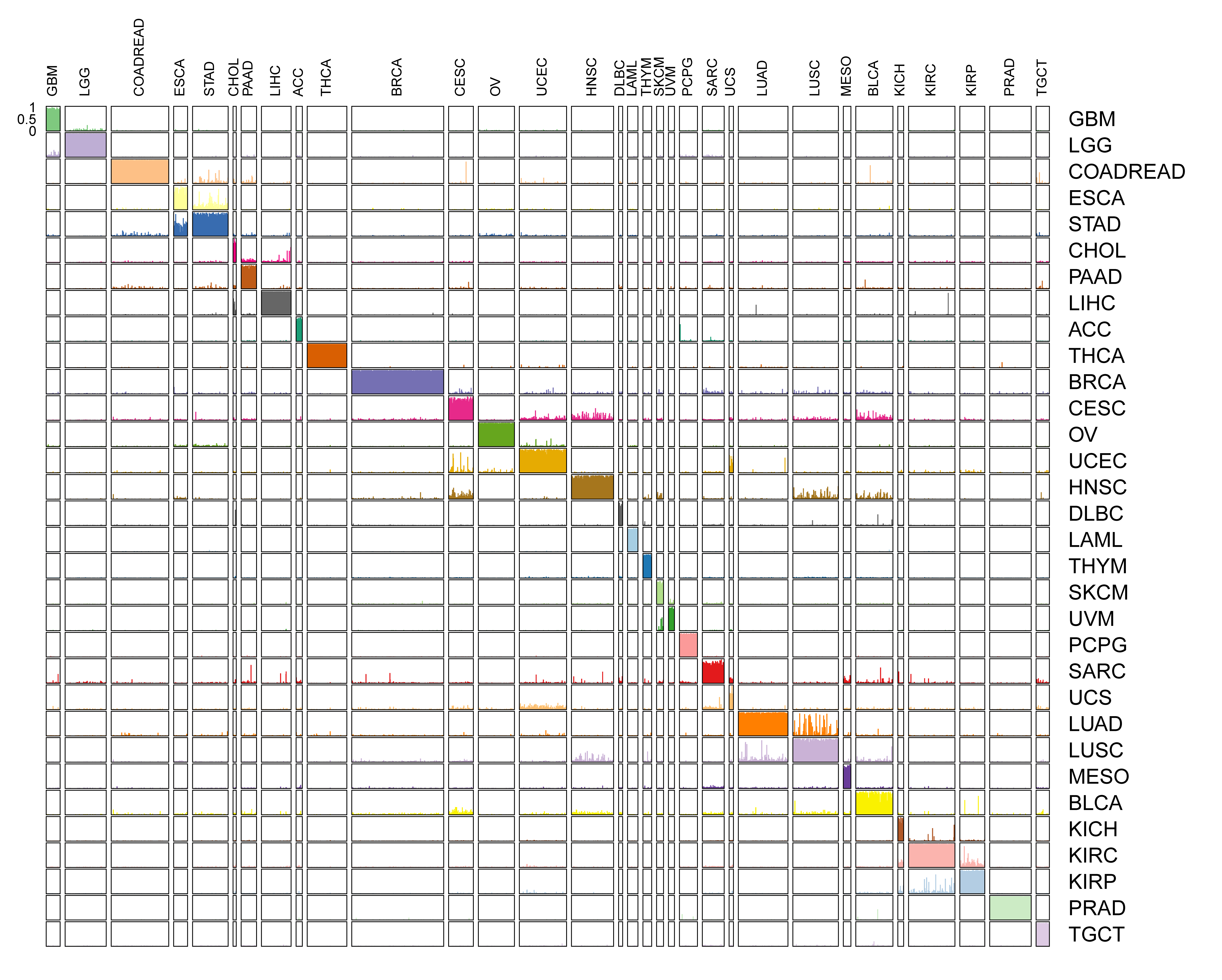


**Pan-Cancer TCGA prediction score on 5044 test samples.** RF Single Sample predictor (accuracy 0.965, mean balanced accuracy 0.972). Prediction uncertainties were primarily within organ systems, or between samples with squamous transdifferentiation.

**References**

Bernardo,C. et al. (2019) Molecular pathology of the luminal class of urothelial tumors. J Pathol, 249, 308–318.

Kamoun,A. et al. (2020) A Consensus Molecular Classification of Muscle-invasive Bladder Cancer. Eur Urol, 77, 420–433.

Mariathasan,S. et al. (2018) TGFβ attenuates tumour response to PD-L1 blockade by contributing to exclusion of T cells. Nature, 554, 544–548.

Marzouka,N.-A.-D. et al. (2018) A validation and extended description of the Lund taxonomy for urothelial carcinoma using the TCGA cohort. Sci Rep, 8, 3737.

Sjödahl,G. et al. (2017) Molecular classification of urothelial carcinoma: global mRNA classification versus tumour-cell phenotype classification. J Pathol, 242, 113–125.

Yoshihara K, Shahmoradgoli M, Martínez E, Vegesna R, Kim H, Torres-Garcia W, Treviño V, Shen H, Laird PW, Levine DA, Carter SL, Getz G, Stemke-Hale K, Mills GB, Verhaak RG. Inferring tumour purity and stromal and immune cell admixture from expression data. Nat Commun. 2013;4:2612.
